# High light intensity plays a major role in emergence of population level variation in *Arabidopsis thaliana* along an altitudinal gradient

**DOI:** 10.1038/srep26160

**Published:** 2016-05-23

**Authors:** Antariksh Tyagi, Amrita Yadav, Abhinandan Mani Tripathi, Sribash Roy

**Affiliations:** 1 Genetics and Molecular Biology Division, CSIR-National Botanical Research Institute, Lucknow, 226001, India; 2 Academy of Scientific and Innovative Research (AcSIR), Anusandhan Bhawan, 2 Rafi Marg, New Delhi, 110 001, India

## Abstract

Environmental conditions play an important role in the emergence of genetic variations in natural populations. We identified genome-wide patterns of nucleotide variations in the coding regions of natural *Arabidopsis thaliana* populations. These populations originated from 700 m to 3400 m a.m.s.l. in the Western Himalaya. Using a pooled RNA-Seq approach, we identified the local and global level population-specific SNPs. The biological functions of the SNP-containing genes were primarily related to the high light intensity prevalent at high-altitude regions. The novel SNPs identified in these genes might have arisen *de novo* in these populations. In another approach, the F_ST_s of SNP-containing genes were correlated with the corresponding climatic factors. ‘Radiation in the growing season’ was the only environmental factor found to be strongly correlated with the gene-level F_ST_s. In both the approaches, the high light intensity was identified as the primary abiotic stress associated with the variations in these populations. The differential gene expression analysis between field and controlled condition grown plants also showed high light intensity as the primary abiotic stress, particularly for the high altitude populations. Our results provide a genome-wide perspective of nucleotide variations in populations along altitudinal gradient and their putative role in emergence of these variations.

The study of genetic variations that impart selective advantages to an organism in its natural habitat is one of the principal goals of ecological genomics. It aims at integrating the genomic changes with the environmental conditions to which an organism is exposed^1^. This environment-driven selection is one of the most important factors behind population differentiation which functions by imposing variable selection pressure among the populations of climatically different regions. Patterns of local adaptation are expected to emerge when selection is spatially variable and strong enough as compared to other acting evolutionary forces such as genetic drift, mutation and migration^2^. The natural genetic variations between the populations of a species are important resources for adaptation to their respective natural environment and to cope with the changing climatic conditions^3^. Over a period of time these variations, in a geographically isolated population, may even lead to speciation^4^,^5^.

Identification of the SNP loci under selection allows understanding the process of the emergence of among-population variations. The correlations between F_ST_s and environmental variables is recently being applied to identify signatures of natural selection^3^,^4^. The advantage of this approach is that it leads to a more straightforward evidence of selection in response to the local climate. Additionally, it also yields fewer false positives^6^ as compared to other approaches such as the outlier approach^7^.

The genetic variations in a population can be classified as shared and population specific (or private) SNPs^8^. The population-specific variants are considered to be recent in their origin as compared to that are shared with other populations^9^ and thus, may have originated *de novo* after colonization of a location. Although the *de novo* mutations are known to be largely deleterious, but if advantageous they can sweep through the population^4^. Recently, NGS of pooled individuals from a population is being applied for the discovery of SNPs and to determine their allele frequency in populations^3^,^10^. This method provides a cost effective alternative to the sequencing of a large number of individuals from a population^11^. Although this method is being primarily applied with whole genome pools, recently, transcriptome pools have also been used for such studies and found to be reliable in case of designs with a large number of individuals in the pool^10^. Moreover, from an evolutionary point of view coding sequences are the key genomic regions to look for the signatures of selection, as they directly influence the protein function^12^. However, selection may also act on SNPs in non-coding regions such as promoters, enhancers or small RNAs where they affect gene expression.

In addition to genetic variations, plants respond and adapt to environmental conditions through plastic changes in their phenotype. Differential gene expression of abiotic stress related genes may help the plants to adapt to the external environment^13^. Modulation of gene expression has a central role in the persistence of an organism under short- and long-term environmental changes^14^. Conversely, an observation of differential gene expression of abiotic stress related genes can indicate a corresponding difference in environmental conditions of treatment groups.

*Arabidopsis thaliana* is an established model in the field of plant molecular genetics^15^. Moreover, the species has also been used for local adaptation studies^16^,^17^. *A. thaliana* populations originating from different altitudes are particularly suitable for studying selection pressures imposed by climatic variables due to the rapid changes in environmental conditions over short geographical distances^18^. Changes in altitude cause variations in atmospheric temperature, pressure and solar light intensity^7^,^19^. These natural abiotic stresses are also known to cause several phenotypic changes in the highland populations^18^,^20^.

The *A. thaliana* populations of Indian West Himalaya inhabit a unique mountainous habitat which ranges from subtropical to temperate climate zones. These populations follow a summer-annual life history^20^. Previously, these populations were morphologically^20^ and genetically^21^ characterized by our group. In the present study, we performed pooled RNA-sequence (RNA-Seq) of four natural populations from four different altitudes (700 m a.m.s.l. to 3400 m a.m.s.l.). Specifically, we asked, what are the SNPs specific to these populations at local and global level? What are the population-specific SNP-containing genes among the populations? What are the SNP-containing genes strongly associated with climatic variables? Are the functions of these genes related to the high-altitude stresses? Additionally, we performed differential gene expression analysis to identify the differences in the transcriptional response of populations between field and controlled conditions. These differences are expected to indicate the abiotic stresses experienced by the populations in the field conditions. Using the two different approaches we attempt to identify the genetic mechanisms accounting for among-population patterns of variations which might have emerged in these populations due to selection under different environmental conditions.

## Results

### Quality and mapping of RNA-Seq reads

**Figure 1:**
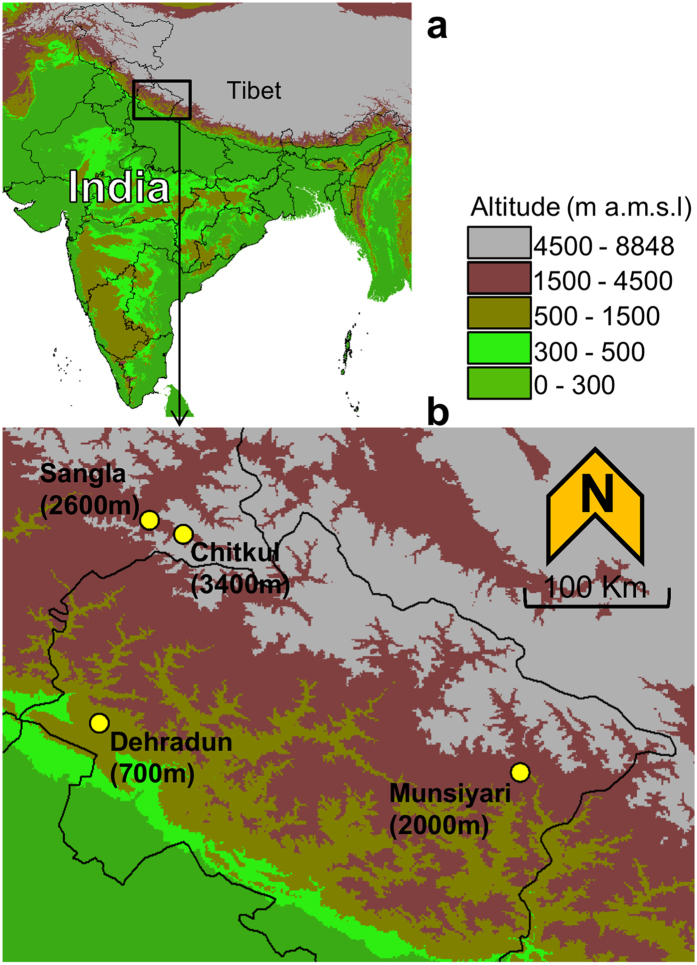
Geographical locations of the sample collection sites. (**a**) Elevation map of India and adjoining region (**b**) Elevation map of West Himalaya showing the sample collection sites (Yellow discs). Figures in parentheses indicate altitude in m a.m.s.l. (meters above mean sea level). See Supplementary Table S1 for the details of geographical co-ordinates, habitat and growing season of the four populations. The open access map was obtained from DIVA-GIS (http://www.diva-gis.org/Data).

Four *A. thaliana* populations were collected from different altitudinal regions of the West Himalaya (Fig. 1 and Supplementary Table S1). The number of paired-end reads, generated from the cDNA libraries of these populations varied from 21.5 m (San, FD) to 34.3 m (Deh, FD). The results of read quality and mapping are shown in Supplementary Table S2.

### Calling of SNPs and their patterns in population

The SNP coverage (against reference genome) of the FD and CC grown plants and that of their combined dataset is shown in Supplementary Figure S1. A total of 340258 SNPs were identified in the four populations. 11.21% of these were of synonymous, and 7.38% were non-synonymous amino acid variants. The various patterns of SNPs were consistent in each population (Supplementary Table S3). There was a significant decrease in the SNP density in populations with the increase in altitude (Pearson’s r = 0.82, p < 0.001). The Transition/Transversion ratio (1.37) was consistent among all the four populations.

**Figure 2:**
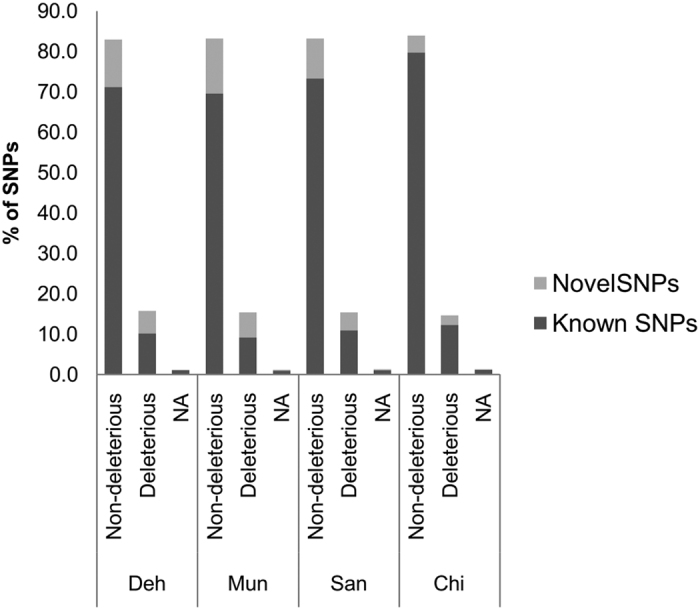
Percentage of non-deleterious and deleterious SNPs (including known and novel) in the four populations. NA indicates the SNPs which did not fall in either of the two categories. Count of SNPs; Deh = 40484, Mun = 40742, San = 40860, Chi = 28610.

In all the populations a large proportion (83%) of the amino acid changes were of the non-deleterious type and only 14.7–15.7% were of the deleterious type. The rest of the amino acid changes did not fall in either of the categories (Fig. 2). In the non-deleterious category, the percentage of known changes varied from 83.6% (Mun) to 95% in (Chi). Whereas, in the deleterious category, the known changes varied from 59.8% (Mun) to 83.5% (Chi) (Fig. 2). It was notable that in all the populations, the percentage of the known changes was higher in the non-deleterious category as compared to that of the deleterious category.

A large proportion of SNP alleles were found to be fixed (allele frequency = 1) in all the four populations (55.9%, 65.1%, 49.1% and 77.3% in Deh, Mun, San and Chi, respectively) (Supplementary Fig. S2a). The percentage frequency distribution pattern of the allele frequencies was highly consistent among the populations when only the shared changes among the four populations were considered (Supplementary Fig. S2b). The percent fixation in the shared SNPs was higher (80%–86.8%) in all the populations as compared to all SNPs. The percentage frequency distribution of the gene level SNP density was found to be highly consistent among the four populations. The highest density was observed in the 400–500 BP bin followed by a gradual decrease (Supplementary Fig. S3). The average allele frequency of all SNP positions ranged from 0.74 (San) to 0.89 (Chi), and that of local-level population-specific SNPs ranged from 0.49 (Deh) to 0.67 (Mun). The allele frequency of global-level population-specific SNPs was also lower than that of all SNPs in all the four populations (Supplementary Fig. S4).

### Functional annotation of local-level population-specific SNP-containing genes

**Figure 3:**
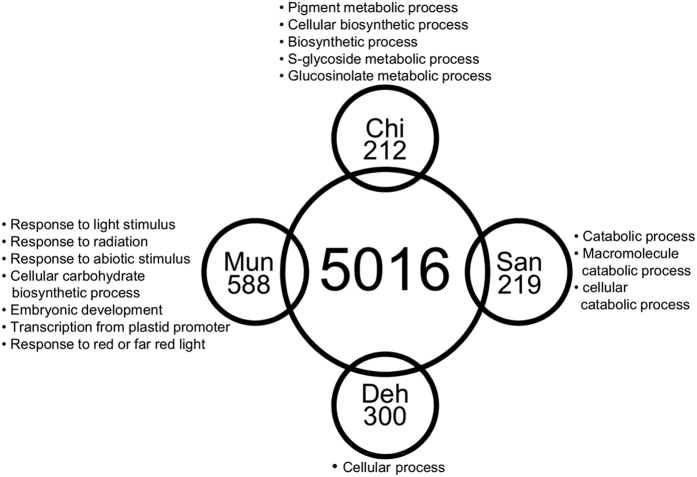
Local-level population-specific SNP-containing genes. Venn diagram showing the count of the local-level population-specific SNP-containing genes in the four populations. The top GO-terms enriched in the four populations are shown. 5016 SNP-containing genes were common among the four populations. See Supplementary data D1 and D2 for the complete GO-term enrichment results and list of genes under the enriched GO-terms.

There were 8188, 8237, 8245 and 6271 genes containing non-deleterious but non-synonymous SNPs in Deh, Mun, San and Chi populations, respectively. Amongst these, 5016 genes were common in all the four populations. There was no difference in the enriched GO-terms (biological processes) among the four populations (Supplementary data D1). Deh, Mun, San and Chi had 300, 558, 219 and 212 local-level population-specific SNP-containing genes respectively (Fig. 3). The GO-term enrichment analysis with local-level population-specific SNP-containing genes resulted in a variety of GO-terms (biological process) enriched in the four populations (Fig. 3 and Supplementary Dataset D2). The Mun and Chi populations showed a significant enrichment of genes related to stress responses. In Mun 85 genes (15.2%) were abiotic stress responsive, out of which 50 (58.8%) were light/radiation responsive (Supplementary Table S4). In Chi ‘pigment metabolic process’ (14 genes) (Supplementary Table S5) and ‘glucosinolate metabolic process’ (9 genes) were found to be significantly enriched GO-terms that could be related to the known abiotic stresses prevailing at high altitudes. Deh showed no GO-term enrichment and San showed no stress responsive GO-term. In San ‘catabolic process’ was the only significantly enriched GO-term, which included two genes related to protection against high light intensity stress *viz.* FAR-RED ELONGATED HYPOCOTYLS 3 (FHY3) and ULTRAVIOLET HYPERSENSITIVE 1 (UVH1). The local-level population-specific SNP-containing genes of the combined dataset of San and Chi did not show any significant enrichment in abiotic stress related categories.

### Functional annotation of global-level population-specific SNP-containing genes

There were 137, 252, 110 and 70 global-level population-specific SNP-containing genes in Deh, Mun, San and Chi respectively. The GO-term enrichment analysis of these genes showed a similar profile of GO-terms as that of local-level population-specific SNP-containing genes (Supplementary Dataset D2). In all the populations, almost half of the genes under the enriched GO-terms were contributed by global-level population-specific SNP-containing genes. Moreover, there was a complete loss in the significance of the enriched GO-terms from the local-level population-specific genes when analyzed without the genes under the enriched GO-terms in global-level population-specific genes. For example, removal of the seven global-level population-specific SNP-containing genes under the ‘pigment metabolic process’ GO-term from the 212 genes of local-level SNP-containing genes resulted in no significantly enriched biological process in Chi.

### F_ST_ Comparisons between populations

The above-stated SNPs were identified against the reference genome of *A. thaliana*. However, to correlate the genomic variations with the climatic variables, the SNPs are required to be called between the populations. Therefore, expressed genome-wide SNPs were called against all pairs of populations and their pairwise gene level F_ST_s were determined.

A total of 29373, 35929 and 36306 SNPs were found in the pairwise comparisons between populations of FD, CC and combined dataset respectively. These SNPs were represented in 13352, 14200 and 9796 genes in the respective datasets. Out of the SNP-containing genes, 7145, 8712 and 8195 were found to have at least one significant (−log_10_ P-value ≥ 1.301) pairwise F_ST_ comparison in the Fischer’s exact test in the respective datasets. We called these genes as ‘highly variable SNP-containing genes’ and were used to correlate with climate data in the partial Mantel test. Out of these highly variable SNP-containing genes in all the tree data sets, ~10% had no known function in the TAIR10 annotations. The remaining genes showed significant enrichment of ‘response to stimulus’ (~32% genes) and ‘response to abiotic stimulus’ (~15% genes) in all the three datasets.

**Figure 4:**
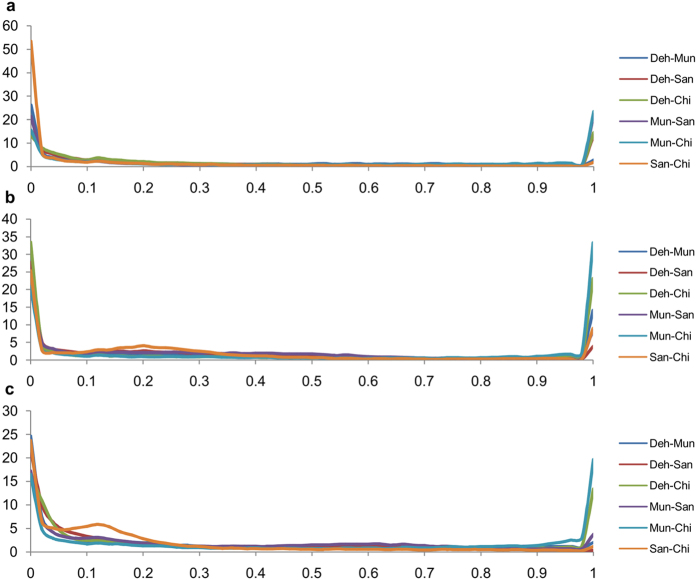
Percentage frequency distribution of pairwise F_ST_s among the four populations in (**a**) Field (FD) (**b**) controlled condition (CC) grown and (**c**) combined dataset of FD and CC populations. The trend lines for the pairwise comparisons of the six population pairs are shown in different colours. x-axis; pairwise F_ST_, y-axis; % frequency.

**Figure 5:**
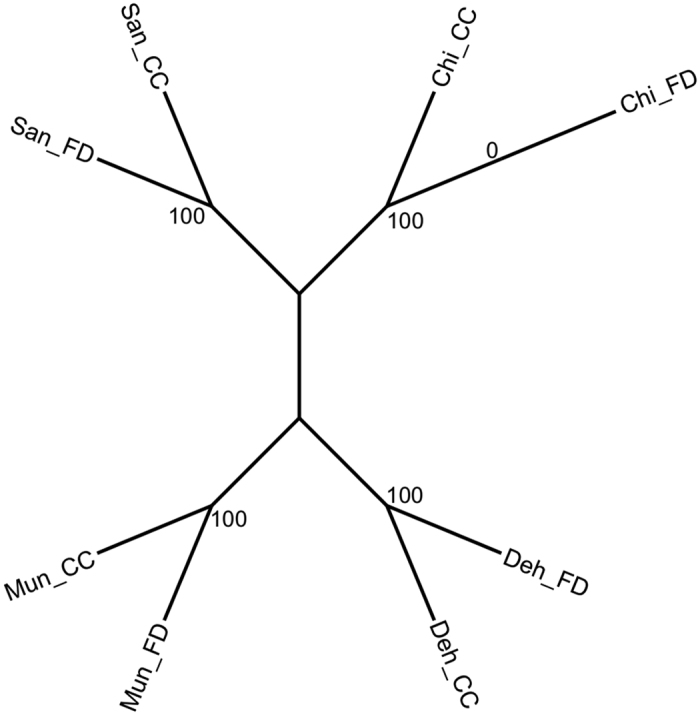
Phylogenetic tree based on SNPs. Majority-rule consensus tree of 500 un-rooted neighbour joining trees constructed using the F_ST_ matrix of the Field (FD) and Controlled Conditions (CC) grown population pools. The pairwise F_ST_ matrix was randomly re-sampled 500 times. Values on the nodes indicate the percentage bootstrap support.

There was a strong correlation between the allele frequencies of the individual FD pools and their CC counterparts with an average F_ST_ of; 0.028 ± 0.0003 SE (Deh), 0.039 ± 0.0004 SE (Mun), 0.035 ± 0.0003 SE (San) and 0.029 ± 0.0005 SE (Chi). The percentage frequency distribution of the F_ST_ values showed that a majority of F_ST_ values lied in the lower range (<0.02) in all the three datasets (Fig. 4). However, in the case of Deh-Chi, Mun-San and Mun-Chi comparisons, a large percentage of F_ST_ values were greater than 0.98. These results indicated that Deh and Chi were the most genetically distinct whereas the San and Chi were the closest among the four populations. The phylogenetic analysis of the four populations also showed that San and Chi were the closest, and Deh and Chi were the most distal populations (Fig. 5).

### Environmental association of SNP-containing genes

**Figure 6:**
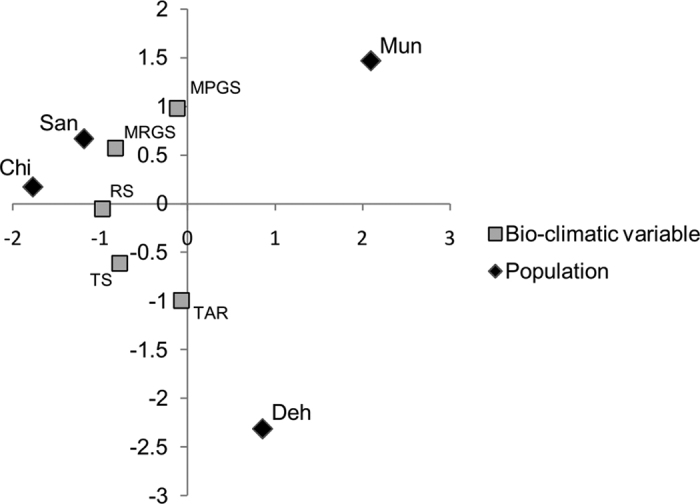
PCoA of Bio-climatic variables. Principle Co-ordinate Analysis (PCoA) of the four populations using five uncorrelated bio-climatic factors; MPGS, mean precipitation in growing season; MRGS, mean radiation in growing season; RS, radiation seasonality; TS, temperature seasonality; TAR, temperature annual range. See Supplementary Fig. S5 for the scree plot of the co-ordinates.

Initially, 26 bio-climatic factors were considered for environmental association analysis with highly differentiated SNP-containing genes. However, only five amongst them were found to be uncorrelated (*r* < 0.8) *viz.* temperature seasonality (TS), temperature annual range (TAR), radiation seasonality (RS), mean precipitation in the growing season (MPGS) and mean radiation in the growing season (MRGS). Further, the bio-climatic variable RS was excluded from analysis as its variation was very small among the four locations (0.19 to 0.22). Results of PCoA analysis of these variables indicated that amongst the four populations, Chitkul and Sangla had the smallest whereas, Chitkul and Dehradun had the largest difference in bio-climatic factors (Fig. 6 and Supplementary Fig. S5).

The 95% quantile of correlation (threshold ‘*r*’) determined by simulation analysis varied for the four bio-climatic variables (Table 1). Overall 380, 700 and 629 genes passed the adopted stringency criteria of having the minimum threshold and a significant P-value in FD, CC and combined datasets. Out of these 54, 120 and 108 genes had a GO-term classification of ‘response to abiotic stress’ in the respective datasets. When the GO-term enrichment analysis was performed for these genes, only the bio-climatic factor ‘mean radiation in growing season’ (MRGS) showed an enrichment in its closely related GO-category i.e. ‘response to radiation’ with 11, 5 and 7 genes in FD, CC and combined datasets respectively (Table 2 and Supplementary data D3, D4, D5). Thus, these genes were identified as having clear association with the local environment.

### Patterns of global gene expression in *Arabidopsis thaliana* populations and validation of differentially expressed genes (DEGs)

The above analyses indicated that the SNP-containing genes were primarily related to light intensity. To further ascertain if light intensity was the major abiotic stress in these locations, we performed differential gene expression (DGE) analysis among these populations. The number of down-regulated unigenes ranged from 37 (Chi-CC vs. Chi-FD) to 105 (Deh-CC vs. Deh-FD) and that of up-regulated ranged from 124 (Deh-CC vs. Deh-FD) to 250 (Chi-CC vs. Chi-FD) (Supplementary Table S6). Amongst the DEGs, on an average 96.1% (92.3 to 100%) unigenes had known functions in the TAIR10 database (Supplementary Table S6). The results of DGE between populations and their GO-term enrichments are provided in Supplementary Table S7a–h. The results indicated that the number of genes and the GO terms related to high light intensity were increasing with altitude.

The DGE values of a few randomly selected genes were further validated by qRT-PCR. The log2 fold-change values resulting from qRT-PCR were highly correlated with that of NGS, with overall of *r* = 0.91 and P-value < 0.0001 (Supplementary Fig. S6). In individual comparisons, the correlation value ranged from 0.85 to 0.98 and was highly significant in all the cases (P-value < 0.0001).

## Discussion

We report genome-wide polymorphisms in the coding region of West Himalayan *Arabidopsis thaliana* populations and their associations with the climatic variables of their natural habitat. The four populations were geographically proximal but colonized diverse habitats with distinct environmental conditions, primarily due to the differences in the altitude of their location. The climatic condition of West Himalayan region range from sub-tropical to temperate^22^. There are several reports on high altitude plant adaptations^5^,^18^,^23^. But, unlike the previous reports, the populations studied here represent a greater altitudinal gradient and a wider climatic range.

The pooling strategy has widely been used for estimation of SNP alleles in populations using NGS^3^,^24^. The strategy has been reported to be as accurate as sequencing individuals^11^,^25^. The pooling strategy has also been employed by using RNA samples (RNA-Seq). Using transcriptome sequencing of 10 bank voles, Konczal *et al*.^26^ showed that the pooled RNA-Seq exhibits accuracy comparable with that of a pooled genome re-sequencing, provided the variation in expression level is accounted for. Moreover, Westram *et al*.^10^ showed that pooled RNA-Seq is reliable for variant allele frequency estimation, given a large number of individuals are included in the pool. RNA-Seq targets only the expressed genes, thereby providing a straightforward understanding of the underlying biological processes^12^. Our strategy of pooling a large number of samples, as well as employing methods such as exclusion of nucleotide positions not present in any of the populations and sub-sampling to an even number of reads helped to minimize the effect of DGE. Moreover, combining the field and controlled condition grown samples also minimized the effect of DGE in addition to providing an increased read depth. Further, the patterns of SNP distribution was found to be highly consistent in terms of depths and alternative frequency distribution indicating a consistently high-quality sequencing across the samples.

We employed a two-way approach to determine the genes that might provide adaptive fitness to populations in a local habitat. First, the population-specific SNPs were identified in each population against the *A. thaliana* reference genome and then the genes containing them were functionally categorized. Our hypothesis was that the population-specific SNPs may have arisen *de novo* in a particular population or may have migrated from a nearby population where they existed as selectively neutral loci in very low and undetectable frequencies^4^. Such polymorphisms may account for the among-population variations and if advantageous in local climate, can cause selective sweeps in populations^27^ and can be linked to the fitness of a population^28^. In the second approach, the gene-level pairwise F_ST_s of the populations were compared with the corresponding pairwise differences between bio-climatic variables. This approach is based on a presumption that an unusually strong correlation between the allele frequency differentiation and environmental variables indicates a potential identification of a candidate locus^29^. Such a strong correlation may also account for the population-level variations. However, such correlation may not always imply the causation for adaptation. The integration of genomic and ecological data may not be straightforward as it is difficult to distinguish between demography and selection as a cause of observed genetic variation^30^.

When all the SNP-containing genes were analyzed, no variation was found in enriched functional categories between the populations. This was due to the large percentage of SNP-containing genes which were shared among the populations (61.2 to 80.0%). A large number of shared SNP-containing genes also indicate a high gene-flow between the populations. However, in the case of local-level population-specific SNP-containing genes, the functional enrichment was found to be markedly different between all the four populations. While the lowest altitude population, Deh did not retrieve any abiotic stress related GO-term, the high altitude populations of Mun and Chi showed enrichment of GO-terms related to high altitude stresses. Importantly, the enriched light-stress responsive genes containing global-level population-specific SNPs appeared to provide a fitness advantage to these populations, as there was observed a complete loss of significance in the GO-term enrichment analyses without these genes. For example, the functional categories enriched in Mun were ‘response to light stimulus’, ‘response to radiation’ and ‘response to red or far red light’. Interestingly, almost half of the observed genes in this functional category contained variants that were global-level population-specific. For example, CULLIN4 (CUL4) is responsible for repression of photomorphogenesis; light harvesting complex of photosystem II 5 (LHCB5) encoding CHLOROPHYLL A/B BINDING PROTEIN CP26 of the antenna system of the photosynthetic apparatus and PHOTOTROPIN 2 (PHOT2) that functions as a blue-light photoreceptor. In Chi, there were seven genes related to pigment metabolism which contained global-level population-specific SNPs. For example, ZETA-CAROTENE ISOMERASE (Z-ISO), a carotene isomerase; CALRETICULIN-3 (CRT-3), which has been linked to anthocyanin accumulation in conjugation with elf18, Chlorophyllase (CLH-1) involved in chlorophyll degradation. Plant pigments such as carotenoids and anthocyanins are known to play an essential role in photoprotection by mechanisms such as scavenging of reactive oxygen species or by absorbing UV radiation^31^. In addition to the pigment metabolism related genes, ‘glucosinolate metabolic process’ was also found to be overrepresented by SNP-containing genes in Chi. Glucosinolates, a class of secondary metabolites in Brassicaceae, have been shown to have a role in plant stress responses including extreme temperatures and high light intensities^32^.

Unlike Mun and Chi, despite being a high altitude population, San did not show any enrichment in the population-specific SNPs containing genes related to the high altitude abiotic stresses. However, two highly characterized genes, FHY3 and UVH1 were found to contain population-specific SNPs in San. The FHY3 transcription factor is a key component in the light signaling pathways such as binding of COP1 promoter in response to UV-B^33^, PHYA signaling pathways^34^ and in the regulation of chlorophyll biosynthesis^35^. UVH1 is a DNA repair gene and a homolog of yeast RAD1. The product of RAD1 is a component of repair-endonucleases and confers resistance to UV radiation by excision repair in response to DNA damage^36^. The non-enrichment of abiotic stress related GO-terms in San could have been due to its geographical proximity to Chi (24 Km downstream on Baspa river). The San population might have got dispersed from Chitkul and due to genetic drift, lost the alleles having the fitness advantage in Chitkul. The milder climatic conditions in Sangla could be a possible explanation for this observation.

Further, the population-specific SNPs had a lower allele frequency as compared to the all-SNPs dataset, suggesting their comparatively recent and *de novo* appearance in these populations^9^. Our earlier study has shown that these populations have a long evolutionary history in the West Himalayan region and the time to their most common ancestor (TMRCA) was found to be about 0.45 million years ago^21^. During this long evolutionary history, the ancestral populations colonized different climatic regions. Subsequently, novel variants might have arisen in response to the selective forces and the beneficial (non-deleterious) among them have swept the populations.

In the second approach, out of the highly correlated SNP-containing genes only 0.75 to 1.37% were found to be strongly correlated with the four uncorrelated bio-climatic factors in the three datasets. Moreover in the combined dataset, the small F_ST_ observed between the field and the controlled condition pools in all the populations confirmed that the variation in gene expression did not confound the allele frequency estimations. The highly stringent procedure allowed us to identify the most relevant SNP-containing genes associated with the four non-redundant bio-climatic variables. These climatic variables have previously been reported to mold plant adaptation^3^,^7^,^16^,^17^. However, out of the four bio-climatic factors only ‘mean radiation in the growing season’ (MRGS) resulted in its closely related GO-term enrichment i.e. ‘response to radiation’, represented by 11, 5 and 7 genes in FD, CC and combined datasets, respectively. The three datasets retrieved the same GO-term for the strongly correlated genes, indicating a role of increasing light intensity in population level variation along the altitudinal gradient. Although the other three bio-climatic variables had strongly correlated genes, none of these retrieved a closely related GO-terms. This was as expected because being an annual with a short life cycle, the climatic conditions during the growing period (three months) has a major effect on the plant. During this period, the solar radiation intensity and its spectrum remains largely unaffected and varies between different altitudes. The effect of increasing light intensity with altitude was also corroborated by the results of DGE analysis. The GO-term enrichment analysis showed that light intensity related genes were primarily expressed in FD samples and, this pattern was more prominent with increasing altitude of populations. Therefore, the enrichment in the correlated GO-category in a radiation-related bio-climatic factor was not surprising. Among the other three selected bio-climatic variables, ‘temperature seasonality’ and ‘temperature annual range’ being annual factors might have a small effect on the development of an annual plant like *A. thaliana*. The ‘mean radiation in growing season’ was the only highly variable bio-climatic factor among the four sites. Thus, the solar radiation of the growing season might play a major role in the population level variation of these populations.

Most of the strongly correlated genes were well characterized. For example, in FD, CONSTITUTIVE PHOTOMORPHOGENIC DWARF (CPD) is known for their role in UV-B acclimation of plants^37^, and FAR-RED ELONGATED HYPOCOTYL 1 (FHY1) is regulated by phytochrome A (phyA) and is involved in balancing the light signaling^38^. In the CC sample, the strongly correlated gene FERULIC ACID 5-HYDROXYLASE 1 (FAH1) is involved in synthesis of sinapate esters, well known to function as plant sunscreens from UV-radiation^39^. In the combined dataset, the strongly correlated gene SUPPRESSOR OF PHYA-105 1 (SPA1) is a regulator of Phytochrome-A. SPA1 interacts with CONSTITUTIVE PHOTOMORPHOGENESIS (COP1) and regulates photomorphogenesis in plants^38^,^40^. Another highly characterized gene, a transcriptional adaptor (ADA2B) is a component of Histone acetyltransferase GCN5 and is involved in plant adaptation to abiotic stresses including high light intensity stress^40^. HIGH CHLOROPHYLL FLUORESCENCE (HCF136) is a stabilizer of PSII and its mutants have been reported to be devoid of PSII activity^41^. A heat shock protein (HSP101) and a heat shock transcription factor (HSFA2) were also among the candidate genes. HSPs are generated in response to high light intensity induced production of ROS^42^ and most of the HSPs are known to respond in high light stresses^43^. It is notable that a heat shock protein (HSP60) of FD sample was also found to be strongly correlated.

Our study may be limited due to the use of interpolated climate data rather than actual field measurements. Being remotely located these sites are presently not equipped with meteorological stations (except in Dehradun). However, interpolated climatic data has been widely used in studies related to genetic diversity, ecological niche modeling, adaptive divergence etc.^3^,^16^,^44^,^45^. The two strategies employed here *viz*. identification of population-specific SNP-containing genes and correlation of F_ST_s with climatic variables retrieved similar kind of GO-terms (i.e. high light intensity). However, the strongly variable genes identified by the two methods were entirely different. This was expected because an SNP having variable allele frequency can never be population-specific and *vice versa*. Another limitation in our study was the use of a small number of populations to detect the environmentally variable genes. Generally, a large number of populations are required to interpret the environment guided variations in the populations such as in Mendez-Vigo *et al*.^5^. However, a comparable number of populations have also been used for identification of genetic variations such as in Fischer *et al*.^3^. Yet, we emphasise on the need to take cautions to interpret these findings in general. However, efforts are being made to sequence more populations from this region.

Our two-way approach led to the identification of genes having a primary biological function related to high light intensity stress. High light intensity is one of the major stresses to which high altitude plants are exposed, as evident from the DGE analysis. Moreover, the environmental factors during the growth period of an annual plant are more important as compared to the annual averages. Thus, the selection of individuals in response to solar radiation stress during growing season of *A. thaliana* might play a major role in the emergence of genetic variation as compared to the annual factors. Further experiments involving functional validation of the genes may provide a deeper insight into their role in population-level variation, particularly in high altitude. Moreover, a number of SNP-containing genes had unknown functions. The SNPs in these genes may also contribute in the emergence of population-level variations in West Himalayan *A. thaliana*.

## Methods

### Collection of samples

Leaf samples were collected from four different populations in the West Himalaya. The populations were named with the first three letters of the name of nearest town or village, *viz.* Dehradun (Deh, 700 m a.m.s.l.), Munsiyari (Mun, 2000 m a.m.s.l.), Sangla (San, 2600 m a.m.s.l.) and Chitkul (Chi, 3400 m a.m.s.l.) (Fig. 1 and Supplementary Table S1). Collections were performed at a stage defined as, plants with first flower open (principal growth stage 6.00)^46^ to plants having no more than 10 green siliques. To minimize the effect of microclimate, areas in each site were selected which maximally represented plants of almost uniform growth and microclimate. A total 50 individuals were randomly selected for each population from five such distinct patches. A minimum distance of two meters was maintained between any two plants. One to two rosette and cauline leaves were collected from each plant and stored in the RNAlater™ solution (Ambion, USA). Seeds of matured plants were collected from each population at the senescence/seed shedding stage. These seeds were grown under controlled conditions of 22 °C, 140 μmol/m^2^/sec light and 16/8 hour day/night cycle. The leaves were collected from the stage as described above for the FD individuals and were stored in RNAlater™ solution for 24 hours before the extraction of total RNA.

### Extraction of total RNA and sequencing

Total RNA was extracted from the leaf tissue of ten plants representing one patch from the field (FD) as well as from the controlled conditions (CC) grown plants using *mir*Vana™ Kit (Ambion, USA). The quantity and quality of the RNA was measured by QIAxpert^TM^ (Qiagen, USA) and 2100 BioAnalyzer™ (Agilent Technologies, USA), respectively. Equal amounts of RNA from each patch of a population were pooled together and considered as representative of the respective populations. The pooled RNA was treated with DNase (Ambion TURBO DNA-free DNase kit). cDNA libraries for the eight pools (four each of FD and CC) were prepared using the TruSeq^TM^ RNA Sample Prep Kit v2. (Illumina, USA) with 1 μg of total RNA., Paired-end sequencing was performed using the Illumina HiSeq 1000 platform.

### Quality control of raw reads, mapping and calling of SNPs against the reference genome

Raw reads with a minimum of 80% bases with a quality score greater than 30 were selected. The filtered reads were mapped against the *A. thaliana* reference genome TAIR10^47^, followed by mapping using Tophat^48^. The Ambiguously mapped reads were filtered using Picard-tools v1.119.

To call the SNPs, the FASTQ formatted sequence files of FD and CC samples from each population were combined. Mapped bam alignment files were used to call SNPs against the *A. thaliana* reference genome (TAIR10) using FreeBayes v0.0.3^49^. The parameters used for calling variants were, ploidy = 200; minimum minor allele frequency = 0.05; minimum coverage = 20. Finally, to remove the biasness due to DGE between different datasets, the nucleotide positions that were not present in any of the populations were excluded. The called SNPs were annotated using the SIFT 4G (siftdb.org) tool. SIFT 4G classifies amino acid variants into synonymous and non-synonymous. Within the non-synonymous variants, it predicts the deleterious and non-deleterious types of amino acid changes. Variants that were not yet reported (as on 31^st^ Mar 2015) in the NCBI dbSNP (ncbi.nlm.nih.gov/SNP) database were identified as novel variants.

### Identification of local- and global-level population-specific SNP-containing genes and their GO-term enrichment analysis

The deleterious and synonymous variants were excluded from further analysis. The remaining non-deleterious but non-synonymous SNPs were classified into two groups of genes. First, the genes containing SNPs that were present in a particular population but absent in other three local populations (local-level population-specific SNP-containing genes); second, such local-level population-specific SNP-containing genes which contained SNPs that were also not reported in the dbSNP (global-level population-specific SNP-containing genes). Our earlier study indicated that the San and Chi populations are phylogenetically very close^21^. Therefore, considering these two as a single population, a dataset of population-specific SNP-containing genes was also included in the analysis. To determine the patterns of SNPs among populations several statistical measures *viz*. the frequency distribution of alternative frequency, the frequency distribution of read depths and average AF of SNPs under local and global population-specific SNPs were estimated.

GO-term enrichment of all the non-synonymous SNP-containing genes and the population-specific SNP-containing genes was performed using AgriGO^50^. The significance for each GO-identifier was computed with the Fisher’s exact test and P-value ≤ 0.05. To adjust for multiple comparisons, significant enrichment was accepted if the corresponding false discovery rate (FDR) was ≤0.05 in Yekutieli’s multi-test adjustment method.

### Analysis of the climate data

Bio-climatic variables of the four locations were derived from the interpolated climatic data. The data was extracted from the WorldClim^51^ database at the highest available resolution of 30″ (second) and collected over a 50 year period (Year, 1950 to 2000). The solar radiation intensity is an important climatic variable along altitudes, but its data is currently not available from the WorldClim database. Therefore, the data for four radiation-related bio-climatic factors was extracted from the CliMond^52^ database at the highest available resolution of 10′ (minute) and collected over a 30 years period (Year, 1961 to 1991). It is important to assess the effect of climatic factors prevailing only during the growing season in these annual populations. Therefore, we derived three additional bio-climatic factors *viz*. mean temperature (MT), mean precipitation (MP) and mean radiation (MR) of the growing season (GS) of three months in the respective locations. Thus, overall 26 bio-climatic variables were considered for the association analysis (Supplementary Table S8).

Pairwise correlation analysis (Pearson’s *r*) was conducted among these 26 bio-climatic variables to exclude the highly correlated factors (*r* ≥ 0.8). To illustrate climatic variations between the sampling locations, Principal Co-ordinate analysis (PCoA) was performed.

### Estimation of the gene-level allele frequency differentiation between populations (F_ST_)

Pairwise F_ST_ analysis of the FD and CC samples was performed using the PoPoolation2 program^53^. The SNPs were called using Samtools followed by the filtering of indels. To minimize the confounding effect of DGE on allele frequency estimation, the allele count data was sub-sampled to an even target coverage of 10 per variant position while excluding upper 2% most highly expressed reads following Westram *et al*.^10^. Random sub-sampling cycles were run in 50 replicates using sub-sampling with replacement strategy. Subsequently, the allele frequency at each position was averaged to get an unambiguous synchronized SNP dataset. F_ST_s at gene level were calculated using the sliding window strategy with a pool size of 100. Fisher’s exact test was performed to test the significance of the F_ST_s. To identify the patterns of F_ST_s between populations, frequency distribution of gene level F_ST_s was determined in bins of 0.01.

The gene-level F_ST_ was also estimated for the combined data of FD and CC samples at an even target coverage of 20 per variant position and pool size of 200, following the above protocol. Further, to determine the effect of DGE on the SNP allele frequency, the position level F_ST_s were determined between individual pools.

### Estimation of overall population differentiation (F_ST_)

The population level F_ST_ between all the pairs of populations in the combined data and individual samples (FD and CC) was calculated using Genepop v4^54^. A majority-ruled (MJ) consensus tree was constructed by random re-sampling the population level F_ST_ matrix 500 times using PHYLIP^55^.

### Correlation between pairwise F_ST_ and Environmental factors

Genes with at least one significant F_ST_ comparison we called as ‘highly variable SNP-containing genes’. There were 7145, 8712 and 8195 highly variable SNP-containing genes in FD, CC and combined dataset respectively. These genes were used to correlate with the five selected environmental factors using the partial Mantel’s test (PMT). The pairwise F_ST_ matrix (D^x^) of a gene was correlated with the distance matrix of four uncorrelated environmental factors (D^y^) individually while controlling for the overall F_ST_ (D^s^, population structure) between the populations by following Fischer *et al*.^3^. The PMT runs of these genes were performed using the parametric Pearson’s *r* correlation in the vegan R package by permuting D^x^ 1000 times.

### Identification of genes strongly correlated with environmental variables

To identify genes with a strong correlation with environmental variables, a three-step stringency cut-off procedure was employed. First, the effect-size ‘*r*’ was determined as a threshold criterion for the correlation values between F_ST_ and bio-climatic variables. F_ST_ values between 0 and 1 were randomly generated to create 7145, 8712 and 8195 simulated matrices corresponding to the same number of highly correlated genes in FD, CC and combined dataset respectively. For this, we first performed PMTs between the simulated pairwise F_ST_ values (D^x^) (random values) and the five environmental factors (D^y^) (real values), while controlling for the population structure using genome-wide pairwise F_ST_ (D^s^) (real values). The 95% quantile of each bio-climatic factor was used as the threshold for *r*. Above this threshold value, a specific SNP locus was considered to be associated with the respective environmental factor. Second, genes having the *r*-values larger than the threshold but insignificant (P-value > 0.05) were discarded. Third, only those genes which fell under ‘response to abiotic stimulus’ GO-term were selected. This allowed us to exclude genes associated with the housekeeping cellular processes, biotic factors and the genes of unknown functions. Finally, GO-term enrichment of genes under each bio-climatic variable was performed. The genes under an enriched GO-term were considered as strongly correlated if the GO-term was closely related to its corresponding bio-climatic factor (Table 1).

### Analysis of DEGs in four populations and their qRT-PCR validation

DGE analysis of four different populations was performed to determine DEGs of FD populations as compared to their respective CC populations (all FD vs. CC). In all the four comparisons the CC population was taken as the denominator for the determination of fold-change in gene expression.

The read counts per unigene were estimated using the HTseq-count program^56^. HTseq-count pre-processes the RNA-Seq data for DGE analysis by counting the number of reads mapped to a gene. The generated read counts were used for the identification of DEGs using the R bioconductor package DESeq^57^. DESeq infers differential signal between a pair of RNA-Seq samples by using a model based on the negative binomial distribution. Finally, DESeq expresses the results in the form of log2 fold-change with their corresponding P-values and false discovery rates (FDR/ Q-value). Unigenes having a minimum of two-fold log2 fold-change value and a Q-value ≤ 0.05 in the pairwise comparisons of the samples were selected for further analysis.

The GO-term enrichment analysis of the significantly expressed unigenes was performed using the Singular Enrichment Analysis (SEA) implemented in the AgriGO online tool. Genes were annotated with the locus identifier information from TAIR10. Significance for each GO-identifier was computed with the Fisher’s exact test. To adjust for multiple comparisons, significant enrichment was accepted if the corresponding Q-value ≤ 0.05 in Yekutieli’s multi-test adjustment method. The lists of GO-terms generated from the results of high-throughput experiments are large and often highly redundant and thus, are difficult to interpret. Therefore, the REVIGO web server^58^ was used to remove this redundancy and summarize the significantly enriched GO-terms in a graphical form which was easier to understand and interpret.

qRT-PCR was performed to validate the gene expression results generated from NGS platform. Ten to fifteen differentially expressed genes (down- and up-regulated) from each sample were randomly selected (Supplementary Table S9). The primers for qRT-PCR were designed using the NCBI primer blast tool^59^ with primer melting temperatures (T_m_) of 57 °C to 63 °C, product size of 90 to 120 bp and a maximum T_m_ difference of 3 °C between the forward and reverse primers. The total RNA from three biologically independent population pools (50 plants each) of FD and CC samples was extracted as described above. Additionally, three technical replicates of each biological replicate were also used. The integrity of RNA was checked by agarose gel electrophoresis. High quality RNA was selected and quantified by using the QIAxpert™ microfluidic UV/VIS spectrophotometer (Qiagen, USA). The RNA was reverse transcribed to cDNA using the SuperScript™ III First-Strand Synthesis Kit (Invitrogen, USA) according to the manufacturer’s protocol. The PCR master mixture was setup by mixing 5 μl DyNAmo Flash SYBR Green (Thermo) (2X), 1 μl cDNA, 1 μl forward primer (5 pm/μl), 1 μl reverse primer (5 pm/μl), and milli-Q water up to 10 μl. qRT-PCR was performed with cycling conditions: initial denaturation at 95 °C for 10 min, followed by denaturation at 95 °C for 20 sec, 40 cycles of annealing and extension together at 60 °C for 60 sec. The amplification reaction was performed using the ABI 7500 real-time PCR system (Applied Biosystems, USA). The threshold cycle (Ct) value for each gene was quantified, normalized by Ct value of internal control gene, *GLYCERALDEHYDE-3-PHOSPHATE DEHYDROGENASE (GADPH)*. The relative expression was calculated using the 2^−ΔΔCt^ method^60^. The relative expression values were then converted to log2 fold-change. The differential expression values from qRT-PCR were correlated with that of NGS using the Pearson’s correlation test.

## Additional Information

**Accession codes:** The RNA-Seq FASTQ files were submitted to NCBI sequence record archive (SRA) with the accession number: SRA347035.

**How to cite this article**: Tyagi, A. *et al*. High light intensity plays a major role in emergence of population level variation in *Arabidopsis thaliana* along an altitudinal gradient. *Sci. Rep.* **6**, 26160; doi: 10.1038/srep26160 (2016).

## Supplementary Material

Supplementary Information

Supplementary Data D1

Supplementary Data D2

Supplementary Data D3

Supplementary Data D4

Supplementary Data D5

## Figures and Tables

**Table 1:** Four un-correlated bio-climatic variables and their 95% effect size cut-off in field (FD), controlled condition (CC) grown and combined populations with their closely related GO-terms.

**Bio-climatic variable**	**effect size** ***r*****(FD, CC, Combined)**	**Closely related GO-terms**
Temperature seasonality (TS)	0.787, 0.799, 0.800	response to temperature stimulus(GO:0009266), response to cold(GO:0009409), response to heat(GO:0009408)
Temperature annual range (TAR)	0.803, 0.808, 0.807	response to temperature stimulus(GO:0009266), response to cold(GO:0009409), response to heat(GO:0009408)
Mean precipitation in growing season (MPGS)	0.813, 0.819, 0.823	response to water(GO:0009415), response to water deprivation(GO:0009414)
Mean radiation in growing season (MRGS)	0.808, 0.808, 0.796	response to radiation(GO:0009314), response to light stimulus(GO:0009416)

**Table 2:** List of genes having strong correlation in the three-step procedure of stringency filtration post environmental correlation tests.

**Sample**	**AGI code**	**Gene name (Abbreviation)**	**NO. SNPs**	**F**_**ST**_ **Deh vs. Mun**	**F**_**ST**_ **Deh vs. San**	**F**_**ST**_ **Deh vs. Chi**	**F**_**ST**_ **Mun vs. San**	**F**_**ST**_ **Mun vs. Chi**	**F**_**ST**_ **San vs. Chi**
FD	AT5G35320	–	10	0.43	0.45	0.48	0.14	0.14	0.21
	AT5G05690	CONSTITUTIVE PHOTOMORPHOGENIC DWARF (CPD)	8	0.60	0.94	1.00	0.19	0.18	0.26
	AT5G05520	–	7	0.49	0.86	0.93	0.16	0.20	0.15
	AT1G27650	(ATU2AF35A)	5	0.08	0.98	1.00	0.89	0.91	0.02
	AT2G37678	FAR-RED ELONGATED HYPOCOTYL 1 (FHY1)	5	0.49	0.67	0.80	0.10	0.15	0.36
	AT2G26930	4-(CYTIDINE 5′-PHOSPHO)-2-C-METHYL-D-ERITHRITOL KINASE (CDPMEK)	4	0.09	0.80	0.83	0.49	0.51	0.01
	AT2G46970	PHYTOCHROME INTERACTING FACTOR 3-LIKE 1 (PIL1)	4	0.45	0.80	0.94	0.55	0.69	0.15
	AT3G23990	HEAT SHOCK PROTEIN 60 (HSP60)	3	0.44	0.66	0.93	0.04	0.22	0.15
	AT4G16780	HOMEOBOX PROTEIN 2 (HB-2)	3	0.27	0.99	1.00	0.42	0.44	0.01
	AT5G06460	UBIQUITIN ACTIVATING ENZYME 2 (UBA 2)	3	0.51	0.83	0.97	0.31	0.55	0.18
	AT1G78420	–	2	0.58	0.72	0.75	0.09	0.12	0.01
CC	AT4G36220	FERULIC ACID 5-HYDROXYLASE 1 (FAH1)	11	0.68	0.66	0.97	0.63	0.97	0.12
	AT4G33650	DYNAMIN-RELATED PROTEIN 3A (DRP3A)	8	0.65	0.40	0.94	0.43	0.99	0.26
	AT1G78420	–	2	0.35	0.23	0.73	0.28	0.83	0.23
	AT4G25570	(ACYB-2)	2	0.03	0.95	1.00	0.84	0.88	0.01
	AT5G20520	WAVY GROWTH 2 (WAV2)	1	0.01	0.05	0.03	0.03	0.01	0.01
Combined	AT5G43470	RECOGNITION OF PERONOSPORA PARASITICA 8 (RPP8)	15	0.26	0.46	0.39	0.26	0.13	0.10
	AT4G16420	HOMOLOG OF YEAST ADA2 2B (ADA2B)	6	0.50	0.70	0.93	0.49	0.77	0.16
	AT1G74310	HEAT SHOCK PROTEIN 101 (HSP101)	4	0.35	0.88	0.80	0.50	0.40	0.03
	AT5G23120	HIGH CHLOROPHYLL FLUORESCENCE 136 (HCF136)	4	0.29	0.59	0.76	0.43	0.61	0.04
	AT2G26150	HEAT SHOCK TRANSCRIPTION FACTOR A2 (HSFA2)	3	0.59	0.99	1.00	0.86	0.87	0.00
	AT2G46340	SUPPRESSOR OF PHYA-105 1 (SPA1)	3	0.58	0.83	0.94	0.54	0.69	0.07
	AT1G15820	LIGHT HARVESTING COMPLEX PHOTOSYSTEM II SUBUNIT 6 (LHCB6)	2	0.0027	0.0056	0.0051	0.0018	0.0005	0.0009

Gene-level pairwise F_ST_ of the four population is shown for the strongly correlated genes in field (FD), controlled condition (CC) grown and combined populations samples.
