# Supplementary material for: High light intensity plays a major role in emergence of population level variation in Arabidopsis thaliana along an altitudinal gradient
Source: Sci Rep. 2016 May 23;6:26160. doi: 10.1038/srep26160 (PMC4876511; doi:10.1038/srep26160)
Supplement: Supplementary Information [file srep26160-s1.pdf]

Supplementary information:

High light intensity plays a major role in emergence of population level variation in *Arabidopsis thaliana* along an altitudinal gradient

Antariksh Tyagi[1], Amrita Yadav[1], Abhinandan Mani Tripathi[1,2] , Sribash Roy[1,2]*

1. Genetics and Molecular Biology Division, CSIR-National Botanical Research Institute, Lucknow, 226001, India

2. Academy of Scientific and Innovative Research (AcSIR), Anusandhan Bhawan, 2 Rafi Marg, New Delhi, 110 001, India

* corresponding author, E-mail: sribashroy@nbri.res.in

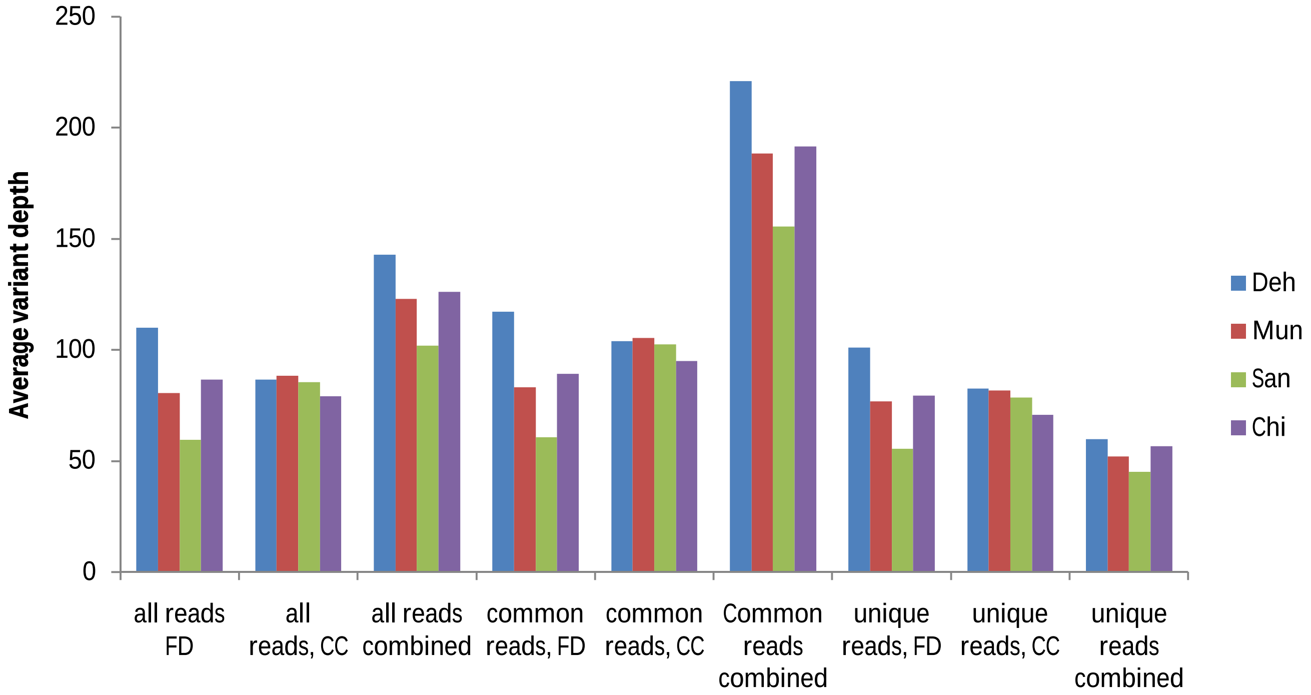

Supplementary Fig. S1. Average coverage of the variant positions of FD and CC samples and combinations thereof.

a

b

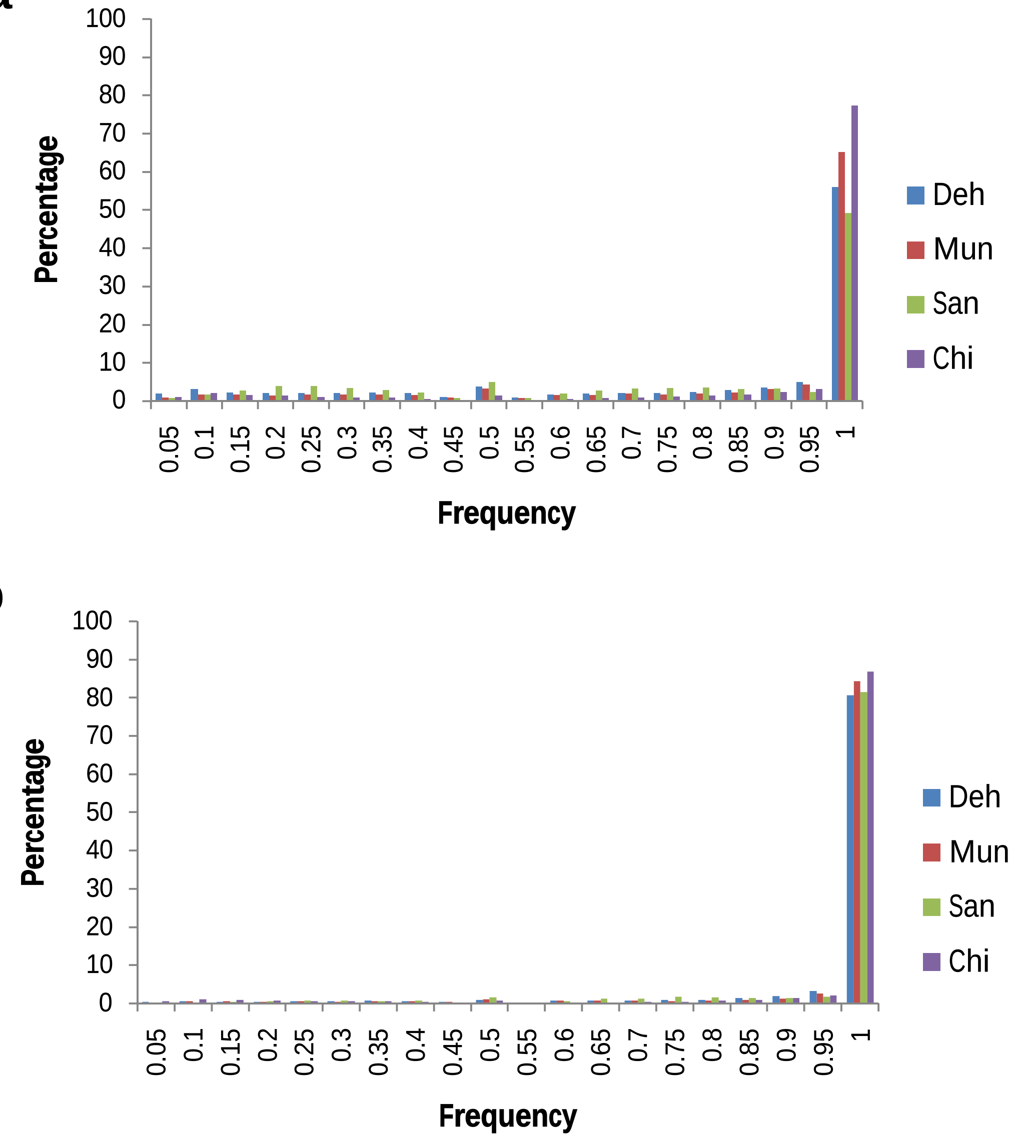

Supplementary Fig. S2. Percentage frequency distribution of the alternative frequencies of SNPs (bin size=0.05). (a) All SNP's in the four populations (b) Shared SNP's between the four populations.

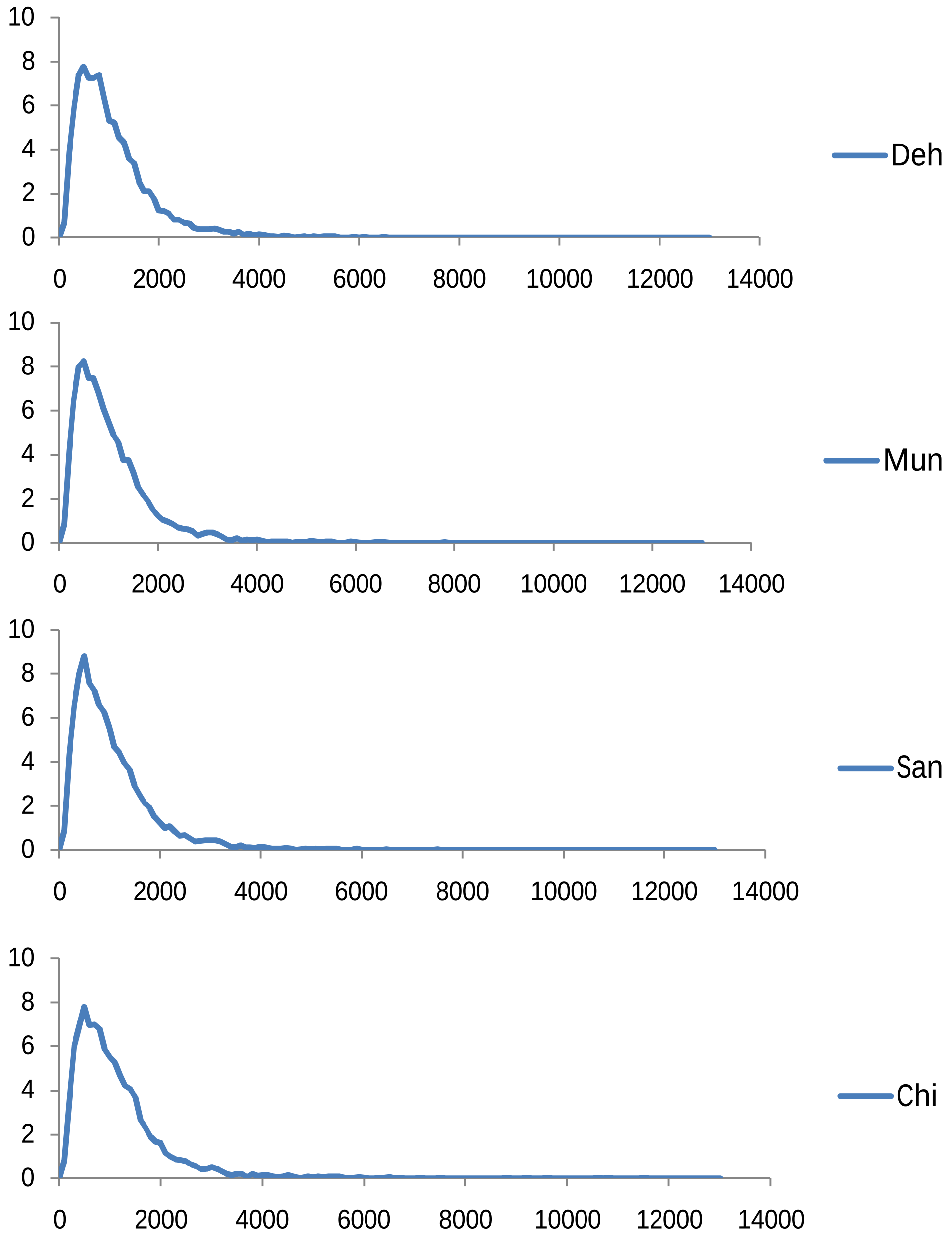

Supplementary Fig. S3. Percentage frequency distribution of read depths in the four studied populations; x-axis Depth Bin, Y-axis: percentage frequency.

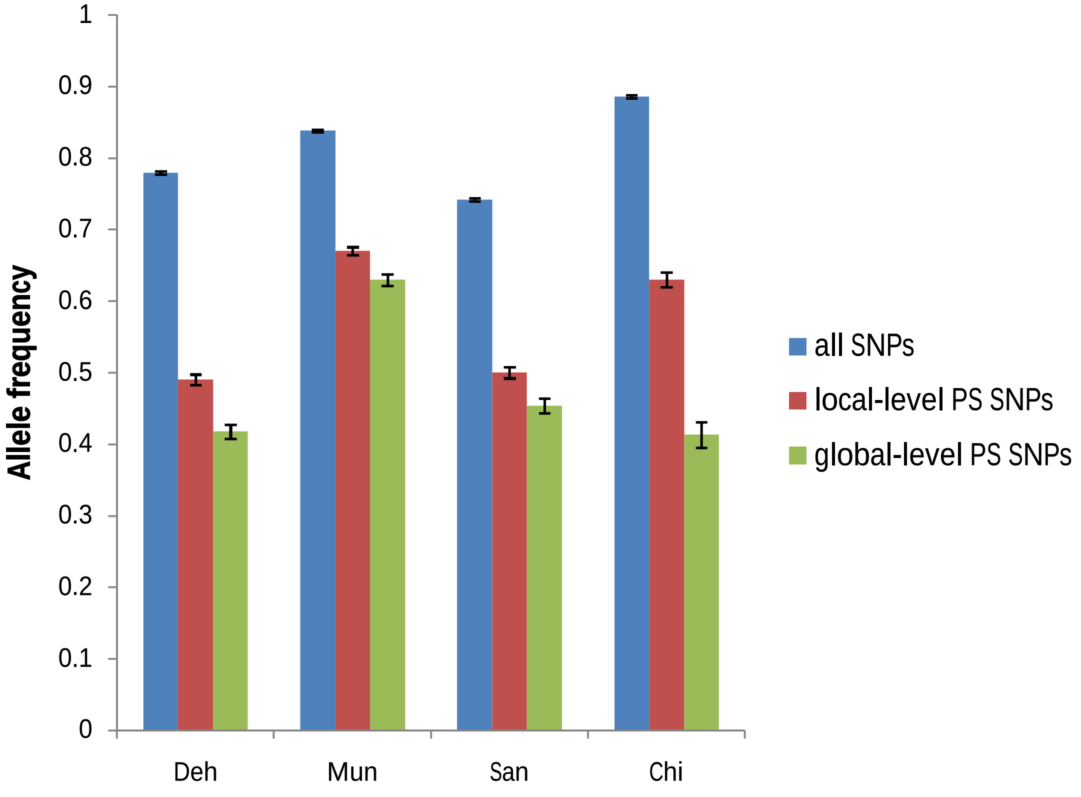

Supplementary Fig. S4. Average allele frequency of non-synonymous SNP positions under the all and the local- and global-level population-specific (PS) categories in the four populations. Error bars indicate standard error of mean (SEM).

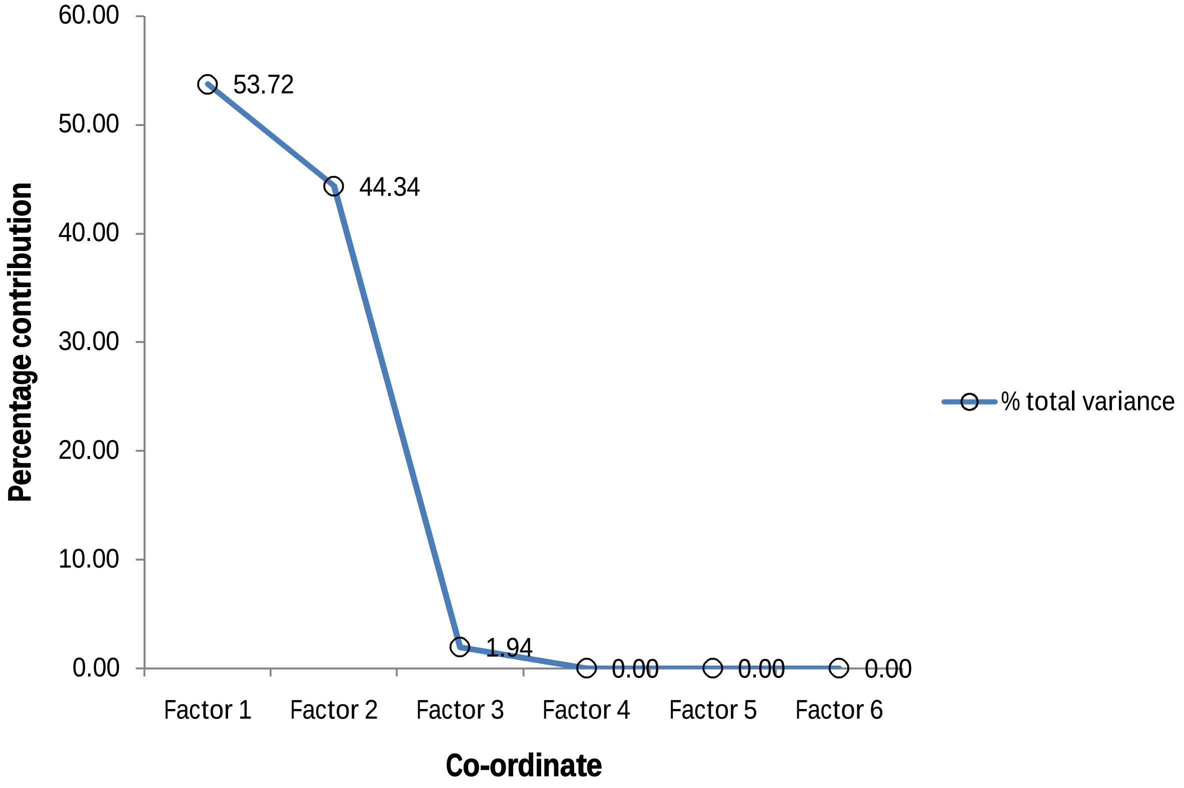

Supplementary Fig. S5. Scree plot. Percentage contributions to the genetic variability of the co-ordinates calculated using principal co-ordinate analysis (PCoA) of four West Himalayan populations using five bio-climatic variables.

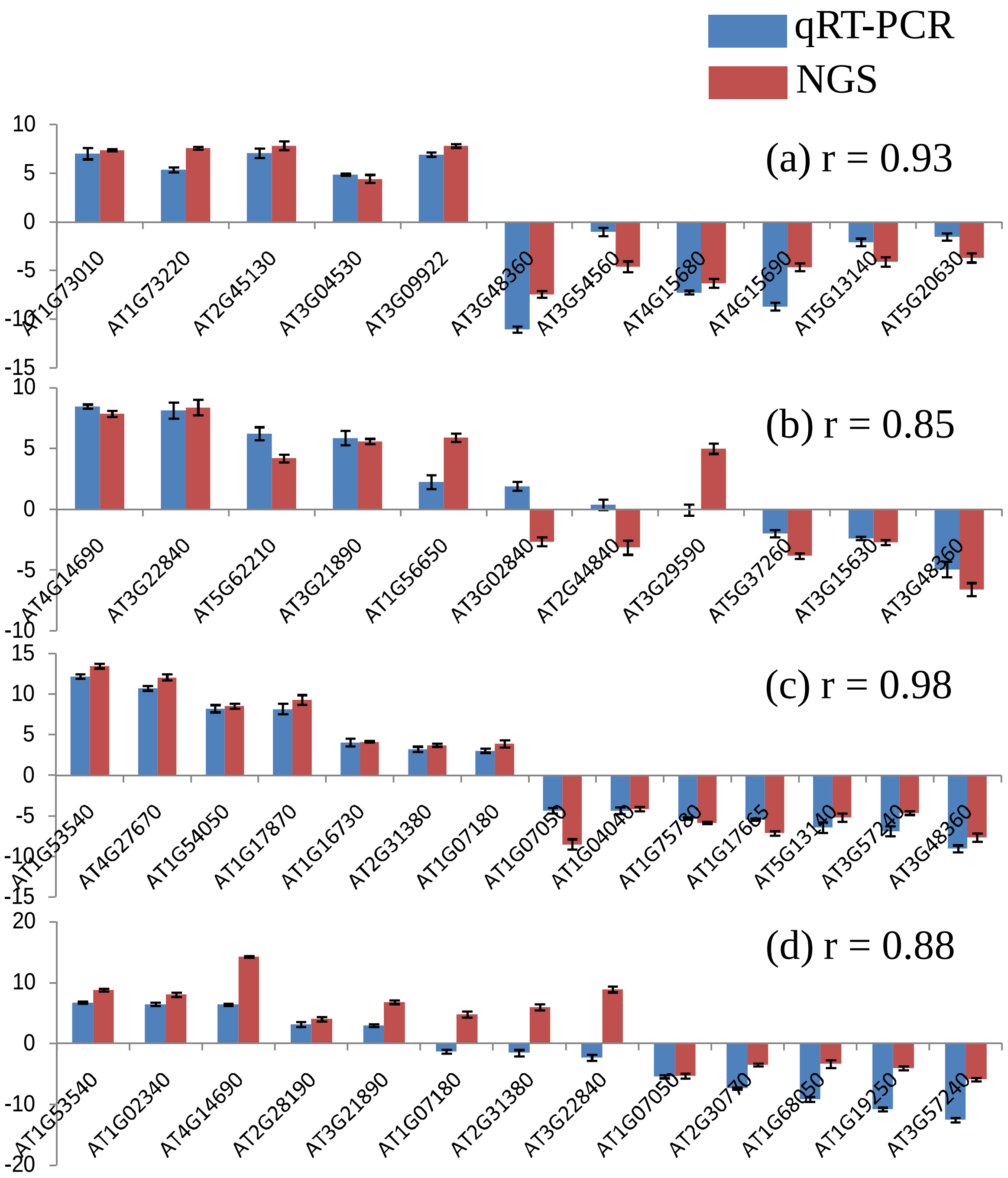

Supplementary Fig. S6. Validation of the gene expression (fold-change) data estimated from NGS using qRT-PCR. Histogram showing log2 fold-change values of qRT-PCR estimated using the $2^{-\Delta\Delta Ct}$ method and that of NGS using normalized red count method. (a) Deh-CC vs Deh-FD (b) Mun-CC vs. Mun-FD (c) San-CC vs. San-FD and (d) Chi-CC vs. Chi-FD Chi-CC. All the correlation (r) values were highly significant (P-value < 0.0001).

Supplementary table S1. The geographical co-ordinates (deg°-min'-sec''), altitude (meters Above Mean Sea Level), habitat and growing season of the populations. See Fig. 1 for the locations on an elevation map.

Location	Population	Latitude	Longitude	Altitude in m a.m.s.l	Habitat	Growing season
Dehradun	Deh	30° 20' 34.1196''	77° 59' 57.5694''	700	Highly disturbed lawn	Dec-Feb
Munsiyari	Mun	30° 4' 50.451''	80° 14' 10.4778''	2000	Undisturbed mountainous slope	Feb-Apr
Sangla	San	31° 25' 22.1592''	78° 15' 54.8598''	2600	Along undisturbed mountainous slope and river valley	Apr-Jun
Chitkul	Chi	31° 21' 0.954''	78° 26' 30.5694''	3400	Undisturbed mountainous slope along river valley	Apr-Jun

Supplementary table S2. Analysis of the transcriptome sequencing of pooled individuals, describing total number of reads, quality filtered reads and mapped reads, number of genes expressed in each sample and their median read count.

Sample	Total Read count	Paired reads after quality filter	Mapped reads		Reads with Multiple alignments		Aligned pairs	Count of Genes expressed	Median read count per gene
			R1	R2	R1	R2			
Deh-FD	34368578	16064175	14564278 (90.7%)	14532026 (90.5%)	131274 (0.9%)	130920 (0.9%)	13916084	22502	174
Mun-FD	27278554	12733468	11272670 (88.5%)	11244246 (88.3%)	141053 (1.3%)	140274 (1.2%)	10753864	22095	148
San-FD	21582974	10017891	7494068 (74.8%)	7476076 (74.6%)	70804 (0.9%)	70588 (0.9%)	7177404	21508	99
Chi-FD	27527390	12867116	11861851 (92.2%)	11833487 (92.0%)	121305 (1.0%)	120755 (1.0%)	11376839	22320	132
Deh-CC	31170808	14563650	13339178 (91.6%)	13302336 (91.3%)	156696 (1.2%)	155973 (1.2%)	12765031	22031	160
Mun-CC	33176190	15496074	14180153 (91.5%)	14159304 (91.4%)	534106 (3.8%)	532060 (3.8%)	13608958	21882	170
San-CC	28776598	13427347	12375311 (92.2%)	12344615 (91.9%)	127064 (1.0%)	126377 (1.0%)	11852892	22202	164
Chi-CC	29825948	13911165	12881601 (92.6%)	12851432 (92.4%)	124580 (1.0%)	124236 (1.0%)	12361333	21692	178

Supplementary table S3. Distributions of the SNPs that were called against the reference genome. The table shows the SNP distribution pattern of in different annotated regions of the genome.

	**Deh**	**Mun**	**San**	**Chi**
SNP count	93815	91146	87341	67956
% Missense	39.09	39.28	38.77	38.7
% Non-sense	0.19	0.18	0.17	0.16
% silent	60.71	60.55	61.06	61.13
% Downstream	37.16	37.18	37.06	36.93
% Upstream	32.78	32.81	32.74	32.79
% Exon	20.16	20.23	20.68	20.03
% Intron	1.7	1.74	1.38	1.72
% 3' UTR	4.42	4.28	4.49	4.53
% 5' UTR	2.54	2.56	2.5	2.7
% Synonymous	11.12	11.18	11.56	11.01
% Non-Synonymous	7.35	7.45	7.55	7.18
Change rate	1275	1312	1370	1760
Ts/Tv	1.37	1.37	1.37	1.367

Supplementary table S4. List of local-level population-specific SNP containing genes in Mun enriched under the GO-term 'response to light stimulus'.

**Locus Identifier**	**Primary Gene Symbol**
AT5G27750	none
AT2G31240	none
AT5G48800	none
AT3G22104	none
AT3G50840	none
AT2G23910	none
AT5G02480	none
AT3G02650	none
AT2G39795	none
AT4G25570	(ACYB-2)
AT3G08010	(ATAB2)
AT5G45340	CYTOCHROME P450, FAMILY 707, SUBFAMILY A, POLYPEPTIDE 3 (CYP707A3)
AT4G31500	CYTOCHROME P450, FAMILY 83, SUBFAMILY B, POLYPEPTIDE 1 (CYP83B1)
AT1G67080	ABSCISIC ACID (ABA)-DEFICIENT 4 (ABA4)
AT1G08250	AROGENATE DEHYDRATASE 6 (ADT6)
AT5G44110	ATP-BINDING CASSETTE A21 (ABCI21)
AT5G23060	CALCIUM SENSING RECEPTOR (CaS)
AT3G01610	CELL DIVISION CYCLE 48C (CDC48C)
AT2G47450	CHAOS (CAO)
AT5G05170	CONSTITUTIVE EXPRESSION OF VSP 1 (CEV1)
AT2G39940	CORONATINE INSENSITIVE 1 (COI1)

AT5G46210	CULLIN4 (CUL4)
AT3G60140	DARK INDUCIBLE 2 (DIN2)
AT4G31160	DDB1-CUL4 ASSOCIATED FACTOR 1 (DCAF1)
AT3G61850	DOF AFFECTING GERMINATION 1 (DAG1)
AT5G35220	ETHYLENE-DEPENDENT GRAVITROPISM-DEFICIENT AND YELLOW-GREEN 1 (EGY1)
AT5G50380	EXOCYST SUBUNIT EXO70 FAMILY PROTEIN F1 (EXO70F1)
AT4G38170	FAR1-RELATED SEQUENCE 9 (FRS9)
AT3G09350	FES1A (Fes1A)
AT4G10340	LIGHT HARVESTING COMPLEX OF PHOTOSYSTEM II 5 (LHCB5)
AT1G17420	LIPOXYGENASE 3 (LOX3)
AT3G02570	MATERNAL EFFECT EMBRYO ARREST 31 (MEE31)
AT4G31010	MITOCHONDRIAL CAF-LIKE SPLICING FACTOR 1 (MCSF1)
AT1G37130	NITRATE REDUCTASE 2 (NIA2)
AT3G55370	OBF-BINDING PROTEIN 3 (OBP3)
AT2G35720	ORIENTATION UNDER VERY LOW FLUENCES OF LIGHT 1 (OWL1)
AT5G58140	PHOTOTROPIN 2 (PHOT2)
AT3G62090	PHYTOCHROME INTERACTING FACTOR 3-LIKE 2 (PIL2)
AT3G48100	RESPONSE REGULATOR 5 (RR5)
AT3G08640	RETICULATA-RELATED 3 (RER3)
AT3G09600	REVEILLE 8 (RVE8)
AT2G39460	RIBOSOMAL PROTEIN L23AA (RPL23AA)
AT2G31190	ROOT UV-B SENSITIVE 2 (RUS2)
AT4G35770	SENESCENCE 1 (SEN1)
AT4G31120	SHK1 BINDING PROTEIN 1 (SKB1)
AT3G61220	SHORT-CHAIN DEHYDROGENASE/REDUCTASE 1 (SDR1)
AT5G13960	SU(VAR)3-9 HOMOLOG 4 (SUVH4)
AT3G47990	SUGAR-INSENSITIVE 3 (SIS3)
AT5G42270	VARIEGATED 1 (VAR1)
AT5G44740	Y-FAMILY DNA POLYMERASE H (POLH)

Supplementary table S5. List of local-level population-specific SNP containing genes in Chi enriched under the GO-term 'pigment metabolic process'.

**Locus Identifier**	**Primary Gene Symbol**
AT2G14910	none
AT4G18440	none
AT1G36280	none
AT1G10830	15-CIS-ZETA-CAROTENE ISOMERASE (Z-ISO)
AT1G08450	CALRETICULIN 3 (CRT3)
AT1G19670	CHLOROPHYLLASE 1 (CLH1)
AT1G76730	CLUSTERS OF ORTHOLOGOUS GROUP 212 (COG0212)
AT2G21330	FRUCTOSE-BISPHOSPHATE ALDOLASE 1 (FBA1)
AT3G29350	HISTIDINE-CONTAINING PHOSPHOTRANSMITTER 2 (AHP2)

AT1G09530	PHYTOCHROME INTERACTING FACTOR 3 (PIF3)
AT1G06570	PHYTOENE DESATURATION 1 (PDS1)
AT1G32990	PLASTID RIBOSOMAL PROTEIN L11 (PRPL11)
AT1G09830	PURINE BIOSYNTHESIS 2 (PUR2)
AT2G36790	UDP-GLUCOSYL TRANSFERASE 73C6 (UGT73C6)

Supplementary table S6. Number of down- and up-regulated unigenes in the four comparison Sets along with the number of genes with known functions in the TAIR10 database.

comparison	No. down-regulated unigenes	down-regulated unigenes with known functions	number of up-regulated unigenes	up-regulated unigenes with known functions
Deh-CC vs. Deh-FD	105	105	124	119
Mun-CC vs. Mun-FD	71	68	185	183
San-CC vs. San-FD	65	60	160	157
Chi-CC vs. Chi-FD	37	35	250	245

Supplementary table S7: GO-term enrichment of differentially expressed genes. Results of REVIGO analysis of the significantly enriched GO-terms identified using AgriGO online tool. The number of differentially expressed unigenes and the total number of genes under a GO-term are mentioned. The table is sorted according to ascending log 10 P-value of GO-term enrichment.

(a) Deh-CC vs. Deh-FD: Down-regulated						
GO-term ID	Description	Number of unigenes	Number of genes in background	log10 p-value	uniqueness	dispensability
GO:0042592	homeostatic process	14	488	-5.3372	0.828	0.0
GO:0009308	amine metabolic process	16	1034	-3.5229	0.88	0.0
GO:0009414	response to water deprivation	10	416	-3.3665	0.631	0.0
GO:0050896	response to stimulus	43	6292	-3.2218	0.971	0.0
GO:0042180	cellular ketone metabolic process	19	2123	-2.1367	0.808	0.056
GO:0071669	plant-type cell wall organization or biogenesis	8	473	-2.0088	0.843	0.053
GO:0006790	sulfur compound metabolic process	9	683	-1.6576	0.898	0.074
GO:0006725	cellular aromatic compound metabolic process	11	1022	-1.5086	0.875	0.137
(b) Deh-CC vs. Deh-FD: Up-regulated						
GO:0006664	glycolipid metabolic process	14	116	-13.0915	0.75	0.0
GO:0016036	cellular response to phosphate starvation	14	169	-11.1739	0.705	0.121
GO:0050896	response to stimulus	62	6292	-9.8861	0.983	0.0
GO:0009718	anthocyanin-containing compound biosynthetic process	9	63	-8.9208	0.766	0.148
GO:0009812	flavonoid metabolic process	11	251	-6.2676	0.924	0.057
GO:0006820	anion transport	10	350	-4.2291	0.851	0.083
GO:0023052	signaling	24	2376	-3.1805	0.98	0.0
GO:0006725	cellular aromatic compound metabolic process	13	1022	-2.3468	0.864	0.108
(c) Mun-CC vs. Mun-FD: Down-regulated						
GO:0050896	response to stimulus	41	6292	-8	0.948	0.0
GO:0006950	response to stress	32	4089	-7.3098	0.593	0.333
GO:0009743	response to carbohydrate	15	812	-6.7212	0.575	0.0
GO:0009611	response to wounding	7	340	-2.7959	0.66	0.236
GO:0007623	circadian rhythm	5	171	-2.3979	0.933	0.0
GO:0048511	rhythmic process	5	171	-2.3979	0.933	0.0
GO:0042180	cellular ketone metabolic process	14	2123	-1.8239	0.766	0.0
GO:0009066	aspartate family amino acid metabolic process	5	278	-1.7212	0.686	0.32
GO:0019725	cellular homeostasis	5	328	-1.5086	0.86	0.126
(d) Mun-CC vs. Mun-FD: Up-regulated						
GO:0010224	response to UV-B	17	104	-14.7212	0.669	0.0
GO:0046283	anthocyanin-containing compound metabolic process	16	87	-14.6198	0.804	0.0
GO:0009812	flavonoid metabolic process	20	251	-12.699	0.938	0.054
GO:0009698	phenylpropanoid metabolic process	21	405	-10.1805	0.828	0.151
GO:0050896	response to stimulus	84	6292	-9.8539	0.978	0.0

GO:0042440	pigment metabolic process	19	361	-9.2924	0.849	0.175
GO:0006575	cellular modified amino acid metabolic process	23	714	-7.585	0.784	0.159
GO:0006725	cellular aromatic compound metabolic process	25	1022	-6.1487	0.92	0.029
GO:0015837	amine transport	10	272	-3.301	0.837	0.054
GO:0051179	localization	39	3819	-1.3468	0.975	0.0
(e) San-CC vs. San-FD: Down-regulated						
GO:0007623	circadian rhythm	13	171	-13.4685	0.952	0.0
GO:0048511	rhythmic process	13	171	-13.4685	0.952	0.0
GO:0043481	anthocyanin accumulation in tissues in response to UV light	6	113	-4.7212	0.448	0.0
GO:0043473	pigmentation	6	113	-4.7212	0.881	0.066
GO:0009825	multidimensional cell growth	5	111	-3.4437	0.781	0.065
GO:0050896	response to stimulus	29	6292	-3.2924	0.963	0.0
GO:0010817	regulation of hormone levels	7	456	-2.6021	0.895	0.0
GO:0071555	cell wall organization	8	613	-2.6021	0.912	0.0
GO:0005976	polysaccharide metabolic process	7	712	-1.699	0.948	0.0
GO:0071554	cell wall organization or biogenesis	8	963	-1.6778	0.93	0.058
(f)San-CC vs. San-FD: Up-regulated						
GO:0009644	response to high light intensity	35	224	-34.7696	0.619	0.0
GO:0006457	protein folding	31	339	-24.8239	0.899	0.0
GO:0033517	myo-inositol hexakisphosphate metabolic process	14	65	-15	0.807	0.037
GO:0050896	response to stimulus	83	6292	-14.3279	0.978	0.0
GO:0008152	metabolic process	101	13633	-3.0088	0.996	0.0
GO:0048518	positive regulation of biological process	15	896	-2.3768	0.842	0.0
GO:0007623	circadian rhythm	6	171	-2.0315	0.975	0.0
GO:0048511	rhythmic process	6	171	-2.0315	0.975	0.0
GO:0015994	chlorophyll metabolic process	6	189	-1.8861	0.906	0.046
(g) Chi-CC vs. Chi-FD: Down-regulated						
GO:0007623	circadian rhythm	8	171	-8	0.953	0.0
GO:0048511	rhythmic process	26	14419	-8	0.953	0.0
GO:0050832	defense response to fungus	8	171	-3.2147	0.565	0.0
GO:0051704	multi-organism process	16	6292	-1.9586	0.956	0.0
GO:0002376	immune system process	6	984	-1.5686	0.954	0.0
GO:0065007	biological regulation	9	1820	-1.5528	0.965	0.0
GO:0050896	response to stimulus	6	342	-1.5086	0.964	0.0
GO:0009743	response to carbohydrate	16	6222	-1.3768	0.674	0.254
GO:0009987	cellular process	5	812	-1.3565	0.981	0.0
GO:0008152	metabolic process	25	13633	-1.3372	0.986	0.0
(h) Chi-CC vs. Chi-FD: Up-regulated						
GO:0009416	response to light stimulus	49	1188	-15.5376	0.598	0.0
GO:0010264	myo-inositol hexakisphosphate biosynthetic process	14	65	-11.7696	0.695	0.0

GO:0009813	flavonoid biosynthetic process	17	225	-8.3872	0.854	0.163
GO:0009812	flavonoid metabolic process	17	251	-7.8239	0.935	0.052
GO:0009698	phenylpropanoid metabolic process	19	405	-6.4559	0.815	0.147
GO:0042440	pigment metabolic process	16	361	-5.0555	0.855	0.175
GO:0015824	proline transport	7	74	-3.7212	0.874	0.061
GO:0007623	circadian rhythm	9	171	-3.1549	0.982	0.0
GO:0048511	rhythmic process	9	171	-3.1549	0.982	0.0
GO:0006725	cellular aromatic compound metabolic process	20	1022	-1.8861	0.888	0.093

Supplementary table S8. Interpolated bioclimatic data: 26 bio-climatic variables derived from monthly mean data of the four population sites. The table includes three seasonal variables derived for this study (highlighted in grey).

**Bio-climatic variable**	**Dehradun**	**Munsiyari**	**Sangla**	**Chitkul**
Annual Mean Temperature [1] °K	295.05	287.15	283.85	279.55
Mean Monthly Temperature Range [2] °K	285.15	283.05	282.15	282.75
Isothermality (2/7) (* 100) [3]	907.4751	1162.34	1092.6	1046.198
Temperature Seasonality (STD * 100) [4]	601.7	504	580.7	621
Max Temperature of Warmest Month [5] °K	309.45	298.65	295.55	291.85
Min Temperature of Coldest Month [6] °K	279.35	275.15	270.55	265.55
Temperature Annual Range (5-6) [7]	30.1	23.5	25	26.3
Mean Temperature of Wettest Quarter [8] °K	299.35	291.45	277.15	272.45
Mean Temperature of Driest Quarter [9] °K	287.85	283.85	280.65	276.25
Mean Temperature of Warmest Quarter [10] °K	301.65	292.45	290.15	286.75
Mean Temperature of Coldest Quarter [11] °K	286.95	280.35	276.05	271.55
Annual Precipitation [12] mm	1915	1461	818	911
Precipitation of Wettest Month [13] mm	582	346	115	119
Precipitation of Driest Month [14] mm	15	14	20	20
Precipitation Seasonality (CV) [15]	128	92.6	42.3	41.4
Precipitation of Wettest Quarter [16] mm	1410	864	306	313
Precipitation of Driest Quarter [17] mm	107	101	117	123
Precipitation of Warmest Quarter [18] mm	746	562	158	248
Precipitation of Coldest Quarter [19] mm	131	176	234	255
Annual mean radiation (W m-2)	184.6992	170.3392	173.79	174.1067
Highest weekly radiation (W m-2)	247.53	220.23	229.16	229.48
Lowest weekly radiation (W m-2	122.11	115.93	110.72	111.64
Radiation seasonality (C of V)	0.2076	0.1974	0.2224	0.2198
Mean temperature in growing season °K	290.23	288.19	287.02	282.63
Mean precipitation in growing season mm	40.4	85.2	75.2	77.4
Mean radiation in growing season (W m-2)	153.6167	179.28	223.3933	223.72

Supplementary table S9: List of primers and their sequences that were used for qRT-PCR validation. The gene highlighted in grey is *GADPH* that was used as an internal control.

Gene AGI code	Forward primer 5'-3'	Reverse primer 5'-3'
AT1G02340	GACGGACAAGGTTTCGGTTC	ACCCCCACTGTTGACATCAT
AT1G04040	TGGCTGGTCCAACCTAATGC	ACCCATCACTCCCCAGACTC
AT1G07050	ATCACCAGAAACCTCTGTGGAC	ACACACTTGCTTCTCTTCGC
AT1G07180	GCAATTGGTGACTGTAGCGG	TCCTCCAGCTTTTCCCATCAC
AT1G16730	GCTCGCTGTTATTGTTCCGTC	GGCAACTTTCCGGAGCTTTC
AT1G17665	CTTCCTTTGACCGACTTCGT	GTTTAAGCTGTTGCTGGAGCC
AT1G17870	ACAACAGCTTGGCTCGTGAT	CGAACCCAAAACAGCTCTTCA
AT1G19250	ACTTGTTGCCCGGAAAACCT	CCGGTACACACAACCACGAA
AT1G53540	CGGATTTACCGGGACTGAGG	ACGGTGCCACTTGTCATTCT
AT1G54050	TCGTGGAAGAGGAAGTAGCAA	AGTCCTCTCTTCCTCCACTGT
AT1G56650	TGGCACCAAGTTCCTGTAAGA	TCCTCTCTTGATACTTGGCTTCA
AT1G68050	AGTCTTCACTGGCTATCGTGC	CAACCAATGGGTGACGCCT
AT1G73010	CCCTGCCATCAAATCTGCAC	CCGAGATGTTCAACGATGGTC
AT1G73220	TTCTTGTGGCTGTTCCTTCCA	TCGACCAAACCATGAATCAGCA
AT1G75780	TGAAGGTGCTGAACTTATTGATGC	TCCAAGAGAGTGACATACTTGAAAC
AT2G28190	GGCGTGGCAGAAACAACAAT	CACCCTTTCCGAGGTCATCC
AT2G30770	GTGGATTCCAAGTTCAAAGAAACG	TGATCAGTTCCGTCATCGTCC
AT2G31380	TCCTTTGTGGGCTACCGATG	AGGCTGGTCACCAAACAGAC
AT2G44840	GCAGAAGGGGATGCAGTACA	TCGTAAGTCCCGAGCCAAAC
AT2G45130	GGCGAAATGGTTCTGCTAGT	CCTCCTCTTGTTCGCTTGTC
AT3G02840	CATGCGATAACGAGCAAGGC	ATTGTGTCGCGAGATCCGAA
AT3G04530	TCGTGATGTTTCTCGGCGAT	TGGAGGTTTCCAACGTTCATCA
AT3G09922	CTTTGGCAAGCTTCGGTTCC	GGGAGTGGGTACAACCCAAA
AT3G15630	TTGGCATGTCATCGGAACCT	CCGTGAGACAACATCGCAGA
AT3G21890	GACTCGGCGTTCCTCTGTAG	GCGCGTGAGTTTCTGACAAG
AT3G22840	GGAGTGAGATGCATGGCTGA	GAGGAGATGGTGACTTCGGC
AT3G29590	GTGAACGAGTGGAGACCGAG	GTCACCTCCACGCATCTTCA
AT3G48360	CGACGCCGTTTCGGTTTTTA	CTTCAGCTGGGTGACCATGT
AT3G49620	CGTACGTAAGAGCAAAGCGT	TCCTCAGCCATGTCGAAATCA
AT3G54560	TTGCATTCTCTCGTCGTCGT	CTTCGCAGCTACGAGTCCTT
AT3G57240	TTCCTTCTTCGACACGGCAG	TTGGTAAAGCGCCACGACTT
AT4G14690	TCCTCCTCCACCAGTTAGCA	CGCCACGAATCCAACCATTG
AT4G15680	ACCCATTTCATCCTCGGCG	AGATCACTACCGACTTCTCGGA
AT4G15690	CACACATTTGCATCCTCCGTG	ACTACCGACTTCTCAGAGATCA
AT4G27670	AGGGAACCAAGGATCAAGTGTC	CGCATCGTCCTCATTGGTGA
AT5G13140	TCACCGTAGTCGGAGTTGTC	TGCTGTTTTTGGTGAACTTGCT
AT5G20630	CTTTGCATTTACCGGCCTCG	GCCATTGATGCCTGCGTAAG
AT5G37260	GGCAGAGCTTGGAGACGAAT	ACACCAAAATCGCGAGCAAC
AT5G62210	AGGTCACTATCGCTTTGGGTG	AACCACCTCCTCCACTTAGC
AT1G13440	TTGGTGACAACAGGTCAAGCA	AAACTTGTCGCTCAATGCAATC
